# Multi-omics study and ncRNA regulation of anti-BmNPV in silkworms, *Bombyx mori*: an update

**DOI:** 10.3389/fmicb.2023.1123448

**Published:** 2023-05-18

**Authors:** Yi-Xuan Fan, Vivian Andoh, Liang Chen

**Affiliations:** School of Life Sciences, Jiangsu University, Zhenjiang, China

**Keywords:** *Bombyx mori*, BmNPV, multi-omics, ncRNAs, miRNA, lncRNA, circRNA

## Abstract

*Bombyx mori* silkworm is an important economic insect which has a significant contribution to the improvement of the economy. *Bombyx mori* nucleopolyhedrovirus (BmNPV) is a vitally significant purulent virus that impedes the sustainable and stable development of the silkworm industry, resulting in substantial economic losses. In recent years, with the development of biotechnology, transcriptomics, proteomics, metabolomics, and the related techniques have been used to select BmNPV-resistant genes, proteins, and metabolites. The regulatory networks between viruses and hosts have been gradually clarified with the discovery of ncRNAs, such as miRNA, lncRNA, and circRNA in cells. Thus, this paper aims to highlight the results of current multi-omics and ncRNA studies on BmNPV resistance in the silkworm, providing some references for resistant strategies in the silkworm to BmNPV.

## 1. Introduction

*Bombyx mori* silkworm (hereafter called silkworm) belongs to the family of Bombycidae and the order Lepidoptera which are known to have originated and domesticated in China about 5,000 years ago (Lu et al., 2018). For the past few decades, silkworms have been widely reared in China, Japan, India, and other countries due to their numerous advantages, such as their low cost, convenience, and no ethical issues. The original purpose for rearing silkworms was to obtain silkworm silk. Silkworm production over centuries ago enriched mankind, encouraged art and culture, and was one of the primary forms of globalization during the Silk Road period (Goldsmith et al., 2005). As one of the important economic resources, silkworm silk has been widely used in the traditional textile industries for several years due to its essential properties, such as its pearly luster, excellent biocompatibility, large-scale production, and mechanical performance (Qi et al., 2017). Recently, numerous novel and essential applications of silks have been explored, such as drug delivery (Tsioris et al., 2012), tissue engineering (Kasoju and Bora, 2012), and so on. In addition, silkworm chrysalis and excrement also have medical values in traditional Chinese medicine (Xia et al., 2014; Qi et al., 2017). The silkworm chrysalis is high in protein and other minerals, making it an excellent source of nutrients for humans and feed additives for animals (Zhou et al., 2022). In addition, due to their important biological role, silkworms have also been used as model organisms for studying environmental toxicology, food safety, drug research, and human disease research (Xia et al., 2014; Qi et al., 2017; Andoh et al., 2021).

**Figure 1:**
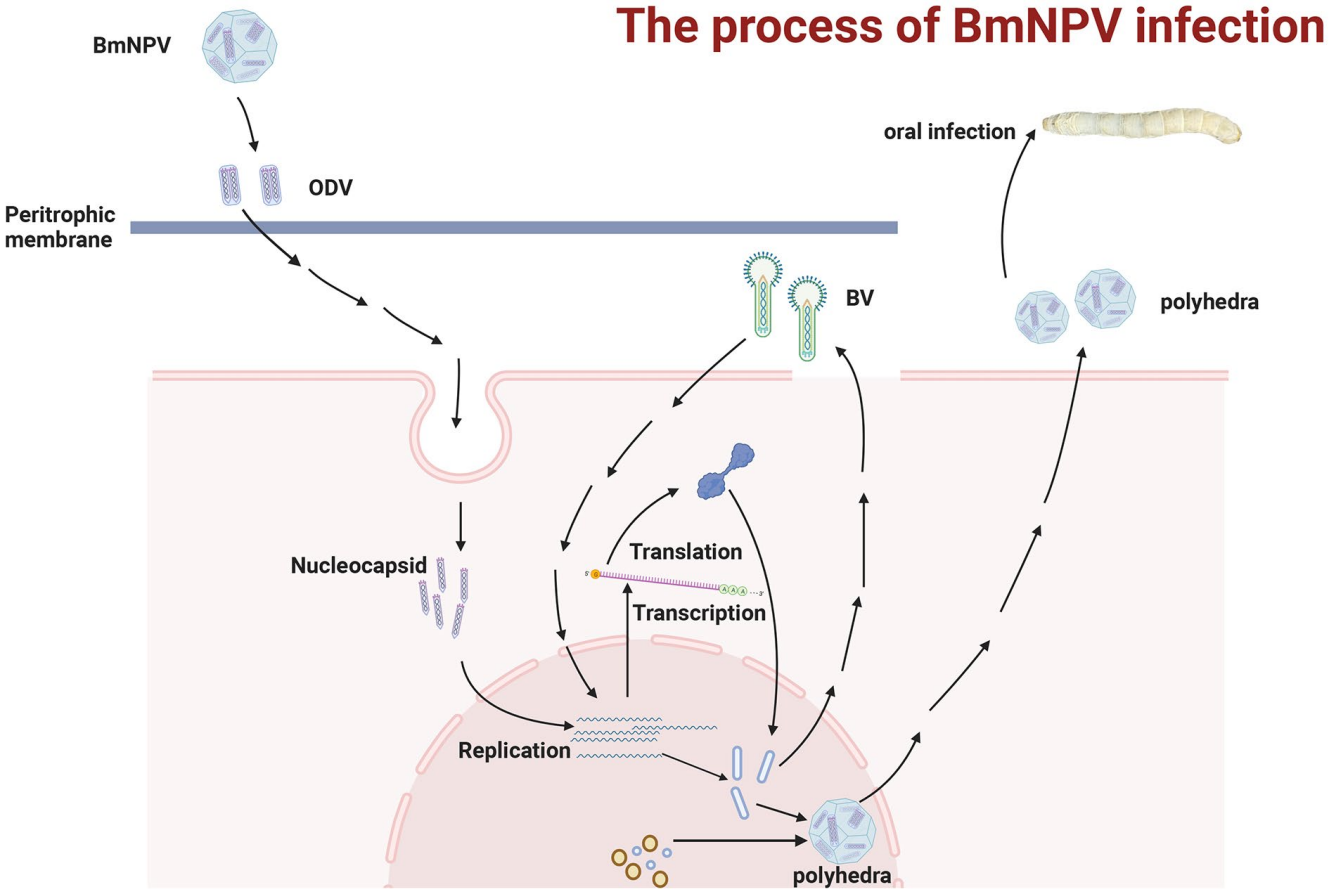
The process of infection with BmNPV in silkworms. After passing through the peritrophic membrane, the viral nucleocapsid fuses with the epithelial cell membrane. It starts DNA replication and translation for the BVs, causing the secondary infection, which spreads in the host cells and tissues. While the ODVs can reassemble to form new viruses that spread among hosts. The figure was created with Biorender.com.

Baculoviruses are double-stranded circular DNA viruses whose genome sizes range from 80 to 180 kb and are known to mainly infect invertebrates, including hundreds of insects (Lacey et al., 2015; van Oers et al., 2015; Jiang, 2021). *Bombyx mori* nucleopolyhedrovirus (BmNPV) belongs to Baculoviridae, and its genome size is about 128 kb (Lacey et al., 2015; van Oers et al., 2015; Jiang, 2021). So far, there are two different BmNPV types identified, including budded virus (BV) and occlusion body-derived virus (ODV) (Gomi et al., 1999; Blissard and Theilmann, 2018; Baci et al., 2022). ODVs mainly infect *B. mori* by oral ingestion and spread the infection from host to host. They pass through the peritrophic membrane to utilize the host to replicate and produce; meanwhile, the BV particles spread between cells and tissues of the infected host, causing systemic infection and resulting in the host’s death (Kondo and Maeda, 1991; Kool et al., 1995). Figure 1 shows the schematic diagram of BmNPV infection and replication within a host. These two types of BmNPV make it challenging to eradicate BmNPV, causing significant loss to sericulture and causing losses to enterprises that rely on the stable development of sericulture. Cocoon losses caused by BmNPV in sericulture production account for more than 60% of all silkworm diseases. However, since the disease has no good treatment, the current stable development of the sericulture industry mainly depends on the regular sterilization of fixed locations (Tang et al., 2017). However, there are also shortcomings and areas for improvement; these ways cannot eradicate the impact of BmNPV on sericulture. Therefore, there is an urgent need for a method that can be used to eradicate BmNPV.

In 1991, more than 340 silkworm strains from the Chinese silkworm germplasm resource bank were identified by Chen’s research group. They discovered that the number of BmNPV-resistant strains showed normal distribution, with the majority being weakly resistant or susceptible and 17 strains exhibiting strong resistance (Chen and Lin, 1991). Subsequently, in 1996, Chen made a resistant identification of the inbred lines, and after 10 years of isolation and purification, the highly resistant strain NB was finally obtained. Genetic analysis of silkworm resistance acted as a single master effector gene control (Hu et al., 2023). These studies laid the theoretical foundation for disease-resistant gene screening and breeding of disease-resistant strains. As early as the 1860s, Hayashiya reported that silkworms fed by the natural mulberry leaf had the natural resistance to BmNPV. A natural red fluorescent substance was then identified in the midgut of silkworms, which was confirmed to be resistant to BmNPV (Hayashiya, 1976; Hayashiya, 1978). Ponnuvel also isolated a protein (Bmlipase-1) in the larval digest that was highly resistant to BmNPV activity and had a lipase activity (Ponnuvel et al., 2003). In the presence of Bmlipase-1, midgut epithelial cells could be protected from ODV invasion during the first stage of infection (Ponnuvel et al., 2003). In the past 10 years, sericulture scientists have been committed to screening silkworm-resistant genes to elucidate the specific mechanism of disease resistance. In 2004, Nakazawa purified the digest of larvae and discovered a protein with a molecular weight of 24, 271 Da that had a strong BmNPV-resistant effect and named it BmSP-2 because it had 94% homology with serine protein (Nakazawa et al., 2004). Proteins like P252, three red fluorescent proteins (RFPs A, B, and C), the silkworm ribosomal protein 3a (Bms3a), the soluble silkworm NADH oxidoreductase-like protein (BmNOX), Bmsop2 were all then found in the midgut or from the midgut juice (Xu et al., 2005; Selot et al., 2007; Pandian et al., 2008; Sunagar et al., 2008; Xu et al., 2008). The results obtained in the study proved that there were differences in BmNPV resistance in different silkworms and provided ideas related to the study of silkworm resistance, setting the stage for numerous subsequent experiments in the silkworms. But the traditional methods to screen each gene or protein in the tissue are incredibly time-consuming and labor-intensive, so it is important to quickly locate essential genes from many genes.

With the rise of multi-omics, such as transcriptomics, proteomics, and metabolomics, it is now possible to rapidly screen the genetic, protein, and metabolite differences between silkworms. These differences are mainly concentrated in species related to heat shock, serine, energy metabolism, and membrane, which has attracted extensive attention (Qin et al., 2012; Wang X.-Y. et al., 2017; Wang X. Y. et al., 2019; He et al., 2021). However, the regulatory mechanism of key resistant genes still needs to be explored while using multi-omics to solve the disadvantages of traditional ways. However, it is worth noting that the RNAs used to carry out biological life activities in living organisms are few, accounting for only 2% of all RNAs in living organisms. In contrast, the rest of the RNAs are used as non-coding RNAs (Feng et al., 2021; Zhao S. et al., 2022). One of these is the constitutive ncRNAs, mainly rRNA and the transfer RNAs (tRNA). In contrast, regulatory non-coding RNAs, as the name implies, can bind to a variety of protein complexes and regulate many important biological functions in the body, including piRNA, siRNA, and other non-coding RNAs. These non-coding RNAs play important regulatory roles in embryonic development, tissue differentiation, signal transduction, organ formation, and other important life activities, and even in developing diseases, viral infections, and antivirals. Regulatory non-coding RNAs can be divided into short, medium, and long non-coding RNAs according to different lengths. Short-stranded noncoding RNAs mainly include piRNA, siRNA, and miRNA (Feng et al., 2021; Xia et al., 2022). The three are similar in length but work in different ways. piRNA mainly interacts with the piwi protein and mediates the silencing of transposon genes (Feng et al., 2021; Xia et al., 2022); siRNA, on the other hand, as double-stranded RNA, binds *in vivo* to become RISC complexes, silences target genes, often imported by foreign genes, may be viruses (Zhao S. et al., 2022). While microRNAs are mainly synthesized in the nucleus, where precursor genes are first cleaved by the Drosha enzyme to form precursor miRNA (pri-miRNA), which is then transported out of the nucleus by Exportin 5, and finally cleaved by the Dicer enzyme in the cytoplasm to form mature miRNA (MacRae et al., 2006), miRNA is also related to RISC complexes, it is a single strand, and binding to the target gene only exists at the 3′ end, siRNA can bind and silence the target gene in all segments of the gene. Long non-coding RNAs include lncRNA and circRNA, etc.

In recent years, miRNAs, lncRNAs, and circRNAs have been the most hotly researched regulatory non-coding RNAs in this category. These RNAs have been discovered to be involved in the resistance to viral invasion and replication in silkworms (Wu et al., 2013; Wu Y. Q. et al., 2016; Hu X. et al., 2018). After miRNA is cleaved by DICER enzymes, an important inhibitor, RISC, mediates the silencing of the gene of interest. lncRNA and circRNA can, in turn, inhibit the expression of miRNAs, thereby promoting the expression and translation of target genes, the regulatory networks between these ncRNAs and mRNA indicate that it is hoped to elucidate the mechanism of disease resistance in silkworms.

Research on disease resistance is now being carried out by scientists worldwide. The current research results show that resistance in silkworms is not only in single tissues but also in a wide range of tissues; genes are involved, and proteins and metabolites were proved to be resistant *in vivo* (Qin et al., 2012; Wang X.-Y. et al., 2017; Wang X. Y. et al., 2019; He et al., 2021). However, fewer studies have addressed the disease-resistant mechanisms in silkworms, and the specific causes of these differences in silkworms are unknown and still need to be discovered. This review summarizes previous studies and explains how these differences may have arisen. We focus on the potential causes of genetic differences and disease resistance through regulatory relationships between various ncRNAs. We explore the future development of ncRNAs in silkworm disease resistance and the possible problems.

## 2. Multi-omics analysis of BmNPV-associated genes

Significant phenotypic differences exist between different lines, distinct in genes, transcripts, proteins, and metabolites (Qin et al., 2012; Wang X.-Y. et al., 2017; Wang X. Y. et al., 2019; He et al., 2021). The use of multi-omics to analyze the differences between other lines and cells, followed by experimental validation, has resulted in considerable advances in screening resistant genes in silkworms over the last decade (Table 1).

### 2.1. Transcriptomics

There are tens of thousands of RNAs in all organisms. Because of the existence of transcriptomics, a large number of RNAs have been found continuously, and this number constantly rises, no matter in which species (Mallory and Shkumatava, 2015). These RNAs play various vital functions in organisms, including immunity, growth and development, behavioral influence, etc. (Mallory and Shkumatava, 2015). Many differences have also been found in the silkworm transcriptome, which are also related to viral infection and resistance (Wang et al., 2015). Among them, the distinction between Dazao-infected and uninfected brains showed 742 differentially expressed genes (DEGs), including 218 upregulated genes and 524 downregulated genes (Wang et al., 2015). Among these differentially expressed genes, 2 genes were significantly related to circadian regulation, 9 genes were related to synaptic transmission, and 3 genes were tightly involved in serum signaling pathways, all of which are speculated to be closely associated with BmNPV infection in *Bombyx mori* (Wang et al., 2015; Abbas et al., 2020). Because irregular movements occur in silkworms in the late stages of infection, these irregular movements might amplify the spread of the polyhedrosis virus among silkworms again (Wang et al., 2015). The differential expression of these brain genes might be due to the impact of the virus, thus indirectly causing their behavioral disorders.

BLAST was used to find differentially expressed genes from previous transcriptomic analysis, *BmβGRP4*, which was found to have an essential inhibitory effect on the replication of BmNPV (Wang J. et al., 2021). RT-qPCR results between different tissues showed that *BmβGRP4* was highly expressed in essential tissues such as the head, skin, midgut, hemolymph, and fat body. At the same time, larvae also showed higher expression compared to adult insects (Wang J. et al., 2021). The infection experiments of P50 showed that *BmβGRP4* had a significant down-regulated expression when BmNPV infect and replicate *in vivo*, while the RNAi of *BmβGRP4* showed a dramatic increase of viral VP39 (The main nucleocapsid protein of BmNPV)at 24–72 hpi (Wang J. et al., 2021). However, overexpression of *BmβGRP4* in BmN cells showed the opposite result, with a significant reduction in the viral replication (Wang J. et al., 2021). The main reason was that *BmβGRP4* could induce upregulation of several apoptosis-related genes, including *BmApaf1*, *BmDredd*, *BmCaspaseNC*, *BmICE,* and *BmCaspase1* to promote virus-induced apoptosis (Wang J. et al., 2021). Previous studies have identified various genes, mainly in the midgut or midgut juice of the silkworm. Still, very few studies have examined the reason for these differences between different tissues. However, studies have shown that the virus replicates in the midgut through the periplasm into the silkworm (Jiang and Xia, 2014).

Nevertheless, numerous studies have concluded that the infection of BmNPV is systemic and affects the whole host body to different degrees, whether it is the brain, midgut, or hemolymph. Therefore, experiments on the entire body needed to be extensively studied to explore the correlation between the differences. In 2006, it was shown that a total of 78, 408 SNPs were identified from the fat body of BmNPV-infected Qiufeng (susceptible strain) compared with the infected Qiufeng N (resistant strain) (Li et al., 2016). In contrast, 56, 786 SNPs were identified in Qiufeng N. Significant nucleic acid sequence differences existed between the two strains (Li et al., 2016). Despite the sequence differences, there were 1,728 DEGs, most of which showed down-regulation in Qiufeng N (Li et al., 2016). Most of the GO enrichment analysis of these DEGs focused on the membrane, metabolism, binding and catalytic activity, cellular processes, and organismal systems. In contrast, KEGG concentrated on oxidative phosphorylation, phagosome, and TCA cycle (Li et al., 2016). These results were similar to an earlier analysis by Xue et al. (2012). Although Xu et al. experimented in the midgut of the silkworm, both experiments involved multiple metabolic pathways and oxidative phosphorylation (Xue et al., 2012; Li et al., 2016). While another analysis of different strains, YeA (resistant strain) and YeB (susceptible strain), showed that after 48 h, 7 potential DEGs between the two groups contained *BmAtlatin-n*, *BmFerHCH*, *BmTHY*, *Bmseroin*, *Bmseroin2*, *BmNHR96,* and *BmSINAL10*. qPCR verified the expression of these DEGs in the midgut and revealed that these genes were differentially expressed between the two after 48 h (Wang X. Y. et al., 2021). All were thought to be potentially involved in the resistant immune response of the silkworm (Wang X. Y. et al., 2021). Although there are commonalities between these studies, further studies are still needed to determine the function of these individual genes.

Resistant genes in domestic silkworms are mainly paternally biased and dominantly inherited. Therefore, resistant genes may have a strong association with sex in silkworms. In a comparative analysis of the highly resistant strain NB and the highly susceptible strain 306 of different sexes, He et al. found that for different sexes of different strains, the number of DEGs either upregulated or downregulated was larger in male NB_V than in female NB_V after infection, while in 306_V, the trend showed the opposite. Eighteen DEGs were uniquely expressed only in NB_V which included 14 upregulated DEGs. The result is consistent with the current view that dominant genes mainly control resistance to BmNPV. Using classical genetics experiments on Cydia pomonella’s baculovirus resistance experiment, Asser-Kaiser proved that a dominant gene controls the resistance gene on the Z chromosome. After 10 years of research, Professor Chen’s team at Jiangsu University found that the BmNPV-resistant NB strain turned out to be a heterozygous population containing three genotypes (++, +−, −). Still, after more than 20 generations of isolation and purification, the laboratory finally obtained a completely homozygous antiviral strain NB (++). Combined with genetic experiments, the resistance of BmNPV in NB might be controlled by a single dominant gene (Hu et al., 2023). These results suggest that a single dominant gene may also control resistance genes in silkworms.

The transcriptomics of different strains and tissues, and even between different sexes, have demonstrated that a large number of DEGs produced in silkworms after infection were inextricably linked to the virus, and these DEGs are concentrated in immune, membrane function and metabolic pathways among different strains in different tissues. Although the large number of candidate DEGs screened by transcriptome may play an important role, there needs to be a more precise explanation for the exact differences in these DEGs. Further detailed experiments are still needed to verify them.

### 2.2. Proteomics

After being infected by BmNPV, the number and expression of proteins in silkworms will significantly change. The protein expression of different silkworms was also different (Liu et al., 2010). Previous studies have also identified multiple resistance-related proteins in the larval midgut (Ponnuvel et al., 2003; Pandian et al., 2008). Proteins might have an essential role in anti-BmNPV. Ten years earlier, a common proteomics analysis of NB ♀, ♂ and 306 ♀, ♂, their direct cross-group (NB ♀, 306 ♂, F1 hybrid) and reciprocal cross-group (306 ♀, NB ♂, F1 hybrid), revealed that 53 and 21 uniquely expressed differential proteins in the two F1 offspring, respectively (Qin et al., 2012). These results also suggest that resistance genes may have a genetic effect and that breeding resistant strains can focus on male hybrids. Because the transcriptome results are the same, the inheritance of resistance genes was more pronounced in male-resistant strains NB (He et al., 2021). Comparative proteome results of both antagonistic and perceptual strains were also shown that only caspase-1 and serine protease were expressed in resistant silkworms but not in the susceptible strain or reciprocal cross group, a result confirmed by WB (Qin et al., 2012). Label-free quantitative proteomic analysis of midgut fluid from infected A35 and P50, the candidate *BmTA* (trypsin, alkaline A) was significantly upregulated in A35 after infection (Zhang S.-Z. et al., 2020). Studies found that a specific concentration of recombinant *BmTA* was associated with the virus co-incubation and had an inhibitory effect on the subsequent replication of ODVs and BVs. The pIZT/V5-His-mCherry vector was used to overexpress *BmTA* in BmN cells. Compared with the control group, the viral VP39 was significantly lower than that of the control group (Zhang S.-Z. et al., 2020).

BC9, a resistant near-isogenic silkworm line generated from resistant line NB and susceptible line 306, the proteomics of its fat bodies after infection showed that the proteome differed after infection. Two of the final selected four proteins involved in energy metabolism, their sequences shared high similarity with phosphoglycerate kinase (PGK) and arginine kinase (AK), respectively (Liu et al., 2010). The remaining protein shared sequence similarity with Pol polyprotein and a novel unidentified protein (Liu et al., 2010). DEPs were mainly involved in energy metabolism since replication of BmNPV requires membrane fusion and DNA replication and translation, all of which require large amounts of energy (Xue et al., 2012). DEPs associated with energy metabolism might then be used by viral replication. Comparative subcellular protein analysis based on the midgut of BC9 and P50 showed that 87 DEPs were screened from them by excluding genetic background (BC9 – vs. P50 –) and individual differences (P50 + vs. P50 –) (Yao et al., 2003; Wang X.-Y. et al., 2017). Energy and protein metabolism, signaling pathways, disease, and transport were predominantly enriched. In particular, protein changes in microsomes were obvious (Wang X.-Y. et al., 2017). The iTRAQ quantitative proteomic showed that between the BC9 and P50, 793 DEPs were found (Yu et al., 2017). After excluding the genetic background and individual differences, 84 total DEPs, 15 cytoskeletons, 12 apoptosis, 8 ubiquitinated, 14 immune, 6 cell signaling regulation, 5 endocytosis-acting, 11 translational, and 14 endopeptidase-associated proteins were selected, all of which are thought to be important in the silkworm (Yu et al., 2017). The fact is that during the infected phase, energy-related, membrane-associated, and molecular functions are significantly different in the host, suggesting the presence of important biological functions.

In recent years, studies of the heat shock protein family have shown the exact resistant function to the virus, but it also played a positive role in the viral replication (Wu et al., 2019b). Wu et al. (2019b) demonstrated that stable replication of BmNPV was affected by inhibition of the HSP90 expression (Shang et al., 2020). Of the 195 DEPs screened in BmN cells inhibited by GA (HSP90 inhibitor), 136 were upregulated DEPs. Expression of immune-related proteins, cellular DNA repair-related proteins, and zinc finger proteins was significantly increased, while kinases were significantly decreased (Wu et al., 2019b). The PPI network showed that 4 heat shock proteins (Hsp12, Hsp20, Hsp23.7, and Hsp70), 5 immune-related proteins, 7 protein kinases, and 10 zinc finger proteins were inextricably linked to HSP90 (Wu et al., 2019b). Further, HSP90 experiments showed its exact positive function to BmNPV (Katsuma, 2021). Other HSP90-inhibited experiments by 17-AAG in BmN cells detected a significant reduction of the viral particles. In contrast, the opposite trend was shown in the control group (treated with a low dose of inhibitor), demonstrating the apparent role of HSP90 in promoting the BmNPV replication (Katsuma, 2021). WB experiments were performed to investigate the specific effect of HSP90 on viral replication. Although HSP90 had no significant effect on the very early gene, it did have significant effects on DBP, BRO, and POLH (early, delayed early, and very late stages of BmNPV, respectively) (Katsuma, 2021).

### 2.3. Metabolomics

The metabolic environment of the silkworm, such as the midgut and fat body, changed dramatically after infection. Studies have shown a dynamic equilibrium between the gut and microbial communities in which microorganisms regulate different biological processes (Ha et al., 2009; Bae et al., 2010; Lee and Hase, 2014). These changes were inextricably linked to the infection of BmNPV. LC–MS/MS analysis indicated that in BmE cells at different infection stages (3 ~ 72 hpi), metabolite pathways were concentrated in ABC transporter, amino acid transport RNA, and purine metabolism signaling pathways (Hua et al., 2021). Cluster analysis was gathered in 5-pyridoxolactone, GPC, 2-OH-Ade, γ-Glu-Cys, and hydroxybutyramide, which all showed a constant upregulation in these periods (Hua et al., 2021). At the same time, only 5-pyridoxolactone strongly inhibited BmNPV replication in the phenotype experiments (Hua et al., 2021). It is shown that metabolites may have a complex resistance mechanism to BmNPV.

Comparing the midgut metabolites of infected and uninfected (Jingsong X Haoyue) silkworms, there were 13 differentially upregulated metabolites, including aspartic acid, asparagine, cysteine, isoleucine, lysine, methionine, phenylalanine, proline, threonine, tyrosine, valine, leucine, and serine (Shi et al., 2021). The authors pointed out that, except for cysteine, isoleucine, proline, and serine, the other nine amino acids were essential for the growth and development of the silkworm (Shi et al., 2021).

The hemolymph metabolites of the YeA (a resistant strain) and YeB (a susceptible strain) found that the expression of trehalose in the YeA continued to rise after infection. Also, the metabolites related to TCA metabolism were actively expressed, and branched-chain amino acids (BCAA) produced irregular fluctuations on the timeline (Wang X. Y. et al., 2019). In another experiment, the microenvironment of the hemolymph, midgut, fat body, and other organs was drastically altered after infection. A total of 296, 215, and 108 DEMs were screened by KEGG and HMDB annotation, and all these changes were thought to be inextricably linked to the BmNPV infection (Wang G. et al., 2021). The above three tissues’ metabolites enrichment results mainly focused on the metabolism of chitin, pyruvate, and glyoxylate, multiple metabolisms of carbohydrates and fatty acids, also with a variety of amino acids such as tryptophan, tyrosine, and phenylalanine (Wang G. et al., 2021). Only tryptophan has been shown to aid in the transformation of indole-3-aldehyde (IAId), indole-3-acetic acid (IAA), and other ligands of the silkworm aryl hydrocarbon receptor (AhR) in the intestines, which can activate a variety of immune cells to be beneficial for immune homeostasis, thereby producing resistance to BmNPV (Gao et al., 2018; Qian et al., 2020). Through metabolomic studies, it was ascertained that metabolites played an important role in silkworm disease resistance; however, problems still need to be solved, including the multiple pathways involved in metabolites and the proportion of key and secondary metabolites. These problems demonstrate the difficulty of studying metabolites in the silkworm; thus, further experiments on disease-resistant mechanisms are required.

BmNPV-resistant genes are present in domestic silkworms and have different roles in different sexes and strains before and after infection (Lu et al., 2018). These differences found by omics such as DEGs found in the transcriptomics involved multiple regulatory pathways, mainly focused on metabolism and membrane transport (He et al., 2021; Katsuma, 2021; Wang X. Y. et al., 2021). Numerous experiments have also shown that these DEGs had resistance to the virus, or among the HSP family screened by proteomics, HSP90 had a facilitative effect on viral replication, while HSP is also regulated by a variety of ncRNAs (Katsuma, 2021; Zhao Z.-M. et al., 2022), other proteins such as serine proteins, VTPases involved in energy metabolism were all shown to differ in the domestic silkworm (Qin et al., 2012; Zhang S.-Z. et al., 2020), suggested a complex mechanism for the role of proteins; moreover, the strong inhibitory effect of 5-pyridolactone on BmNPV (Hua et al., 2021). Also, tryptophan and 9 other amino acids were all proved to be essential to the growth and development of the silkworm and were discovered through metabolomics (Shi et al., 2021).

## 3. Regulation of BmNPV infection in the silkworm by ncRNAs

**Figure 2:**
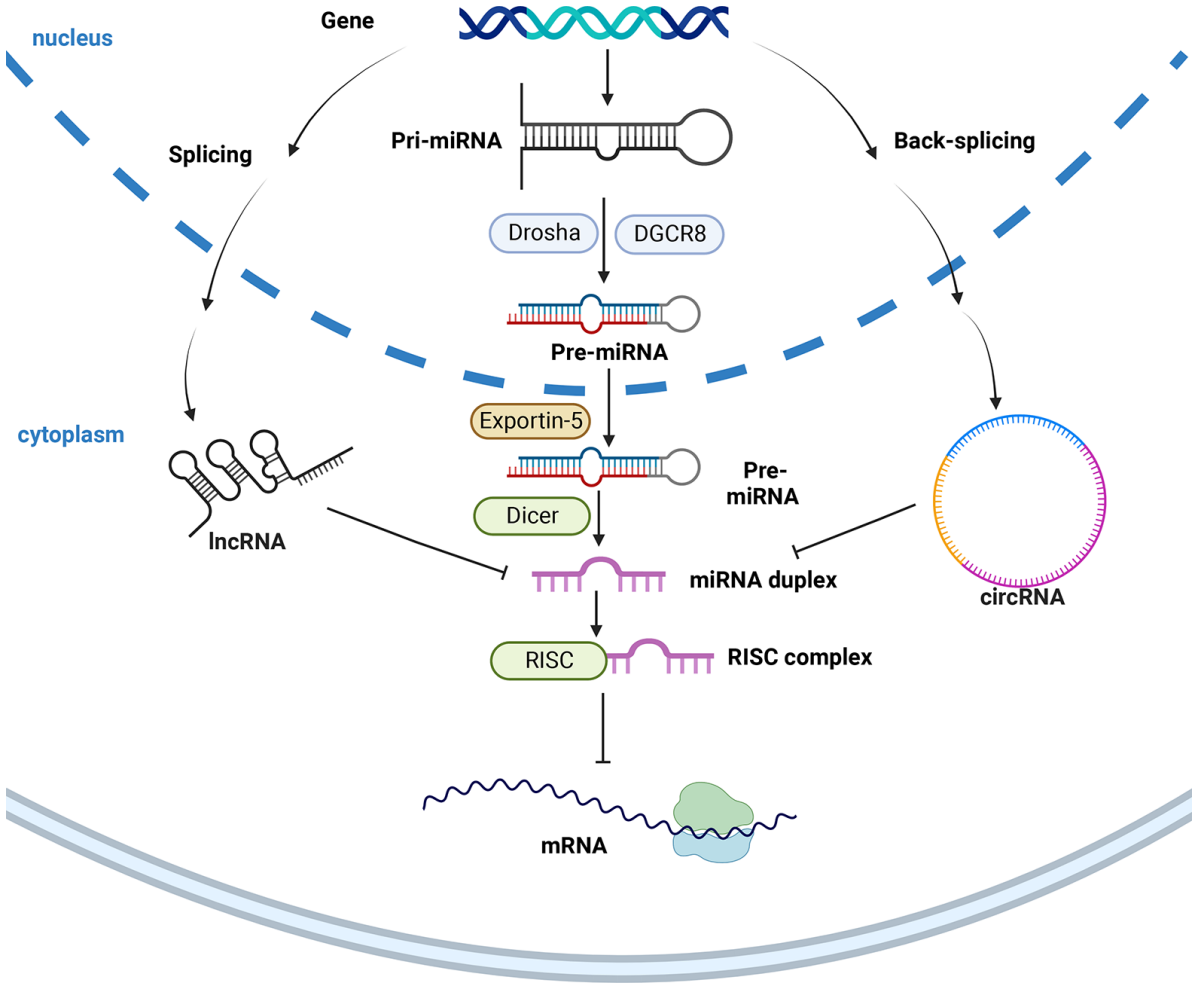
The synthesis of miRNA and the inhibition of ncRNAs. After miRNA is transported from the nucleus by exportin 5, it is cleaved into mature miRNA by Dicer, which is inhibited by lncRNA and circRNA in the cytoplasm, thereby realizing the upstream ncRNA regulation of downstream genes. The figure was created with Biorender.com.

Different types of DEGs, DEPs, and DEMs have all been shown *in vivo* to play important roles in BmNPV resistance. Still, only a few articles have addressed the reasons for the differential expression before and after infection. In recent years, the publication of another class of RNA that does not encode proteins changed the status (Britten and Kohne, 1968; Lander et al., 2001), which included microRNA (miRNA) and long non-coding RNA (lncRNA). In organisms, miRNA, piRNA, and siRNA can silence or inhibit the expression of target genes, respectively, making them attract great interest. While miRNA has a unique regulatory network to regulate its expression, it contains lncRNA and circRNA. Circular RNAs (circRNAs) have also been a hotspot in recent years, mainly characterized by the lack of 5′ end caps and 3′ end tails, which are circular and, therefore, more stable *in vivo*. These RNAs do not directly regulate life activities. Still, they significantly participate in the regulation of mRNA indirectly by circRNA (lncRNA) -miRNA-mRNA, then play a regulatory role for translational effects as well as intermediate metabolites that explains the differential expression between some DEGs, DEPs, and DEMs. Figure 2 shows the miRNA synthesis and the function of ncRNAs to miRNA (Table 2).

**Table 1:** The host genes and substances related to the resistance to BmNPV.

Name	Author	Experimental materials	Expression level	Description
Red fluorescent protein	Hayashiya (1976) and Hayashiya (1978)	Midgut	–	It could interact with the chlorophyll in natural mulberry leaves, and the product had resistance
Bmlipase-1	Ponnuvel et al. (2003)	Midgut	–	Highly homologous with other species, which could protect midgut tissue from viral invasion
BmSP-2	Nakazawa et al. (2004)	Midgut	–	It was found in the digestive juice of silkworms which showed strong disease resistance
P252	Pandian et al. (2008)	Midgut	–	Interacting with chlorophyll also showed the ability of resistance to *Escherichia coli*, Serratia, and other bacteria
RFPs (RFP-A,B,C)	Sunagar et al. (2008)	Midgut	–	The fluorescent moiety showed certain resistance. Lighting is extremely important for the RFPs
Bms3a	Xu et al. (2008)	Midgut	High expression in high resistance strain	Homologous protein s3a in other species has been shown to be involved in the induction of apoptosis
BmNOX	Selot et al. (2007)	Midgut	The resistant line could express higher than the susceptible line	BmNOX affected the virus infectivity, and the protein-coding gene has homology to NADPH oxidoreductase and xanthine dehydrogenase proteins
Bmsop2	Xu et al. (2005)	Midgut	The expression of 306 strain increased after infection with BmNPV	Different expression in silkworms had different resistance levels, especially in susceptible strain after infection which might have related disease resistance
*BMβGRP4*	Wang J. et al. (2021)	Midgut	BMβGRP4 was downregulated in the midgut of the infected P50 strain	*BMβGRP4* could inhibit BmNPV replication through apoptosis
*BmTA*	Zhang S.-Z. et al. (2020)	Midgut	Expression in A35 is higher than strain P50	Overexpression of *BmTA* could inhibit the BmNPV replication
Hsp90	Wu et al. (2019b)	BmN-4 Cell	–	The expression of the Hsp90 could promote the expression of ie-1, and the use of antagonists would lead to the reduction of viral expressed genes
5-pyridoxolactone	Hua et al. (2021)	BmE cell	Expression of BmE continued to rise after virus infection	5-pyridoxolactone, a product of the vitamin B6 metabolic pathway, inhibited viral expression at the initial stage of BmE cell infection

### 3.1. Functions of siRNA and piRNA in anti-BmNPV

As two classes of short-stranded regulatory noncoding RNAs, also known as miRNAs, the study of piRNA and siRNA in BmNPV resistance is equally important. piRNA can use transposon cleavage to influence the expression of target genes, targeting the degradation of target transcripts through PIWI proteins interacting with it. In addition, piRNA can also cope with viral infection (Nie et al., 2013; Xia et al., 2022). Feng et al. identified a large number of piRNAs from the location transposon region in the fat bodies and midgut of the DaZao strain infected with BmNPV. These piRNAs had significant differential expression before and after BmNPV infection; compared with normal tissues, 322 piRNAs were significantly upregulated, and 276 were significantly downregulated in the fat bodies after BmNPV infection. In the midgut, 129 piRNAs were significantly upregulated, and 117 were downregulated considerably, indicating that piRNA has a specific role in silkworms (Feng et al., 2021). In the latest study, the replication of AcMNPV in lepidopteran insect cells sf9 requires the expression of piRNA. Xia et al. found that PIWI-like-1 and PIWI-like-2-3 can inhibit the replication of AcMNPV. At the same time, PIWI-like-4-5 can promote the replication of the virus (Xia et al., 2022).

siRNA is a class of double-strand noncoding RNAs that mainly function as foreign RNA and are, therefore, active in studying viral genes (Zhao et al., 2021). Long-term evolution of viruses has also resulted in the development of RNAi that can evade host immune responses. This includes siRNA, a type of exogenous dsRNA, which is first cleaved and bound to Dcr-2 and to form an RNA-induced silencing complex (RISC) with Ago2. Then the siRNA is cut off a strand, and the remaining vsiRNA plays a corresponding RNA degradation (Zhao et al., 2021). Dcr-2 expression was elevated in silkworms infected with BmNPV, suggesting that siRNA may play a role in BmNPV (Li et al., 2020). In the 2022 study, BmNPV p35 was shown to promote the replication of the virus by inhibiting host siRNA interference pathways (Zhao S. et al., 2022). By comparing BmNPV infection in RNAi-deficient cells, it was found that BmNPV did not show a difference in these cells, demonstrating the importance of siRNA for viral infection (Zhao S. et al., 2022). Further results showed that pinp35 also inhibited the production of vsiRNA and could disrupt the binding of siRNA to Ago2 protein, resulting in the failure of the host RNAi pathway and promotion of the BmNPV replication (Zhao S. et al., 2022).

### 3.2. MicroRNA

Tong et al. (2006) aligned silkworm miRNAs by using mature miRNAs identified from model animals as query sequences, showing that there were 24 miRNAs in the silkworm genome. Wu P. et al. (2016) constructed a miRNA library from the silkworm P50 and applied Solexa sequencing technology to identify 38 differentially expressed miRNAs between infected and uninfected groups (Wu P. et al., 2016). Among the selected differentially expressed miRNAs, *miR-282*, *miR-10-5p*, and *miR-277-5p* were included in the series of genes expressed in cells after BmNPV infection (Wu P. et al., 2016). For the individual miRNA experiment, upregulation of miRNA *bmo-miR-3378* could inhibit the replication of the virus, while the inhibited investigation showed the opposite trend (Jiang, 2019). But the validation of downstream targets is still lacking and needs to be improved because of their non-coding features (Jiang, 2019), resulting in unclear mechanisms. As a non-coding RNA, it remains to be clarified how it causes difficulties in viral replication (Table 2).

**Table 2:** Functions of ncRNAs in resistance of silkworm to disease and viral replication.

Name	Author	Type	Experimental tissue (cell)	Expression level	Description
*Bmo-miR-282*	Jiang (2019)	miRNA	Strain P50	Upregulated in infected strain P50 larvae	The expression level was changed by infection, which was related to virus replication
*Bmo-miR-10-5p*	Jiang (2019)	miRNA	Strain P50	Upregulated in infected strain P50 larvae	Related to the replication of virus
*Bmo-miR-277-5p*	Jiang (2019)	miRNA	Strain P50	Upregulated in infected silkworm P50 larvae	Experiments showed this miRNA can interact with *Dnmt2* which could cause several immune pathways
*Bmo-miR-3378*	Jiang (2019)	miRNA	BmN cell		The overexpressing of *Bmo-miR-3378* significantly reduced the viral amount
*Bmo-miR-390*	Kang et al. (2018)	miRNA	Hemolymph	The expression was significantly reduced after virus infection	Expression changed with the increase of time after infection and showed the ability to downregulate the *cg30 in vitro*
*Bmo-miR-217*	Wang M. L. et al. (2017)	miRNA	Hemolymph	The experimental group was higher than that in the treatment group after infected	Interacting with *lef-1* which could inhibit the *lef-1*
*Bmo-miR-2819*	Wu et al. (2019a)	miRNA	BmN cell	Expression decreased after viral infection in BmN cells	After infection of the virus, the expression of miR-2819 would inhibit virus
*Bmnpv-miR-1*	Singh et al. (2012)	miRNA	BmNPV	–	*Bmnpv-miR-1* indirectly caused decrease in the host own miRNA, which will promote viral replication
*Bmnpv-miR-3*	Singh et al. (2014)	miRNA	BmNPV	–	By regulating P6.9 protein and various viral proteins which could promote virus replication
*BmNPV-miR-415*	Cao et al. (2017)	miRNA	BmNPV	–	Viral miRNA induced host *Bmo-miR-5738* to regulate *TOR2*, and thus regulated viral replication
*KO122AS*	Ishihara et al. (2015)	lncRNA	BmNPV	–	Mutants of *KO122AS* could produce fewer OB virions than wild type
*Lnc_209997*	Lin et al. (2022)	lncRNA	Fat body	The expression of BmNPV was significantly down-regulated after infection	By regulating *miR-275-5p* to promote the replication of virus
*circEgg*	Wang et al. (2020)	circRNA	Strain DaZao	–	Through *bmo-miR-3391* to regulate the acetylase expression

Using high-throughput sequencing technology, two miRNAs named *bmo-miRNA-390* and *bmo-miR-217* were predicted from different *Bombyx mori*. The prediction proved that the above two miRNAs could interact with *cg30* and *lef-1* proteins, respectively (Wang M. L. et al., 2017; Kang et al., 2018). Both of these BmNPV proteins were indispensable in the replication of the BmNPV (Wang M. L. et al., 2017; Kang et al., 2018). By constructing luciferase reporter plasmids containing genes encoding *cg30* and *lef-1* proteins, co-transfection with miRNA mimics into cells to investigate the binding of miRNAs and viral proteins. The fluorescence of the experimental group was weaker than the control group, indicating the interaction between miRNA and viral proteins (Wang M. L. et al., 2017; Kang et al., 2018). Meanwhile, in BmNPV-infected BmN cells, transfection of mimics or inhibitors of both miRNAs revealed that *bmo-miRNA-390* and *bmo-miR-217* could effectively down-regulate the expression of *BmNPV-cg30* and *BmNPV-lef-1* in infected BmN cells, respectively (Wang M. L. et al., 2017; Kang et al., 2018). It affected the infection and replication of BmNPV in *Bombyx mori* (Wang M. L. et al., 2017; Kang et al., 2018). Later, Wu et al. (2019a) identified that *Bombyx mori* miRNA, *bmo-miR-2819* was the target of BmNPV-*ie-1*. BmNPV could reduce the expression of *bmo-miR-2819* to enhance the expression of viral *ie-1*, thus promoting the viral replication (Wu et al., 2019a). Dual luciferase experiments confirmed their interactions between *bmo-miR-2819* and *ie-1*, additional experiments involving overexpression and inhibition of the *bmo-miR-2819* demonstrated that the virus could reduce the expression of *bmo-miR-2819* to enhance viral *ie-1* expression, thus promoting viral replication (Wu et al., 2019a).

In addition to the host miRNAs, viral miRNAs are also significant. Viral miRNAs could regulate host miRNAs and indirectly regulate the expression of the host’s genes or proteins, such as host Ran and *Bmo-miR-5738* (Singh et al., 2012; Cao et al., 2017). Thereby realizing the effect of enhancing the self-replication (Tang et al., 2019). In 2004, miRNAs were first discovered in the Epstein–Barr virus (a herpes virus) (Pfeffer et al., 2004). Subsequently, hundreds of viral miRNAs were identified, including baculovirus and adenovirus. In 2009, Shirina successfully predicted the existence of miRNAs in BmNPV, such as *bmoNPV-miR-1ph* and *bmoNPV-miR-2ph,* using RNAfold (Shirina et al., 2011). Singh also identified the viral miRNAs, like *bmnpv-miR-1* and *bmnpv-miR-3* (Singh et al., 2010), and predicted the targets of these miRNAs, which were all considered to be significantly involved in the insect-pathogen interactions by regulating several replicating genes (Singh et al., 2010). It was observed that *bmnpv-miR-1* could regulate the miRNAs in host cells, thereby regulating the host-virus defense mechanism affected by the host miRNAs (Singh et al., 2012). Though there is no direct evidence that viral miRNAs can directly regulate host miRNAs, in 2012, the mechanism of *bmnpv-miR-1* was proved to affect Ran expression (an essential component of exportin-5 protein) and, in turn, downregulated the expression of *bmnpv-miR-1* which resulted in the reduction of BmNPV replication (Singh et al., 2012). *Bmnpv-miR-3* is also a BmNPV-produced miRNA that could regulate the P6.9 protein and various viral proteins to escape the immune attack of host cells (Singh et al., 2014). In addition to the direct regulation of multiple proteins, viral miRNAs can also indirectly regulate host miRNAs that can, in turn, regulate the protein expression (Cao et al., 2017). *BmNPV-miR-415* was also found that it could down-regulate the expression of *Bmo-miR-5738* in BmN cells and indirectly affect the expression of TOR2 protein, which plays an important role in the growth and development of the host (Cao et al., 2017). Although the reasons for how viruses downregulate host miRNAs have not been elaborated, viral miRNAs could regulate host miRNA transport proteins (Singh et al., 2012). *BmNPV-miR-415* may likely have a similar function, regulating host proteins to affect the expression of host miRNAs and miRNA-regulated proteins (Cao et al., 2017). miRNAs have enriched the content and depth of BmNPV-resistant research. Still, the specific role of viral miRNAs for BmNPV resistance in silkworms requires further experiments to clarify their requirement for virus replication or their role in the regulation of host immune responses and pathways.

### 3.3. Long non-coding RNA

LncRNA is widely found in animals, plants, viruses, and fungi, where they play important roles in epigenetic, transcriptional, and post-transcriptional regulation (Rinn et al., 2007). As a non-coding RNA, its function is as simple as host immunity and viral replication (Fatica and Bozzoni, 2014; Li et al., 2014; Ishihara et al., 2015; Sun and Kraus, 2015; Adelman and Egan, 2017; Zhang et al., 2021). In 2014, Ishihara identified many antisense lncRNAs in the BmNPV genome. There were 15 lncRNAs identified from the sequences of the early and late promoters in BmNPV, and the mutants of these genes were constructed and infected the silkworm (Ishihara et al., 2015). Compared to the ODVs produced by wild types *KO122AS*, the ODVs produced by the mutant *KO122AS*, encoded by the *Bm122* genome, were significantly reduced, showing that the BmNPV lncRNA might have essential roles in promoting viral replication and spread by regulating its gene expression (Ishihara et al., 2015).

Except for the viral lncRNA in the silkworm, Zhang et al. used whole transcriptomic sequencing technology to analyze 4,450 differentially expressed lncRNAs, 66 DEmiRNAs, and 7,448 DEmRNAs in the BmNPV infected and uninfected BmN cells to construct a lncRNA-miRNA-mRNA network. It was then found that these networks focused on the protein hydrolysis and lysosomal pathway, all of which were significantly associated with the immune pathways in silkworms (Zhang S. et al., 2020). Furthermore, the results indicated that after infection with BmNPV, the silkworm could regulate its self-immunity through the lncRNA-miRNA-mRNA pathway (Zhang S. et al., 2020). In the fat body, *Lnc_209997* was significantly down-regulated in BmNPV-infected silkworms, and *Lnc_209997* could also inhibit BmNPV replication by regulating *miR-275-5p* expression (Lin et al., 2022). Therefore, to promote its replication *in vivo*, BmNPV reduced the expression of host *Lnc_209997* and indirectly regulated *miR-275-5p* (Lin et al., 2022). Since these studies, the function of lncRNA is becoming better understood. As ncRNAs, the specific lncRNA-miRNA-mRNA regulatory network suggests that lncRNA may act indirectly through miRNAs to regulate mRNA expression regardless of the non-coding feature of itself (Ahadi, 2021). The subsequent studies of their functions should focus on discovering their target genes, as this can help elucidate the specific functions of lncRNA, thereby elaborating the capability of lncRNA and their regulation of vital silkworm activities.

Yin et al. (2020) constructed a DElncRNA-*bmo-miR-278-3p*-*BmHSC70* regulatory network in GA-treated BmN cells and found that the lncRNA and *miR-278-3p* were responsible for the regulation of *HSP70* expression (Yin et al., 2020). *HSP70*, on the other hand, was found to have possible protein interactions with host HSP90 and may be influenced and regulated by BmNPV (Wu et al., 2019b). Zhao used the exogenous inhibitors of HSP90, geldanamycin (GA), which could inhibit the expression of HSP90, and finally inhibit the replication and proliferation of the virus (Zhao Z.-M. et al., 2022), which was similar to the previous studies carried out by Wu et al. (2019b). However, Zhao detected 29 DEmiRNAs in the cells after GA treatment and constructed a DElncRNA-DEmiRNA-DEmRNA regulatory network (Zhao Z.-M. et al., 2022). This network included 169 DElncRNAs, 12 DEmiRNAs, and 69 DEmRNAs. GO and KEGG analysis of these DEmRNAs showed that these DEmRNAs are mainly involved in ubiquitin-mediated proteolytic, phagosomal, proteasomal, and endocytic pathways (Zhao Z.-M. et al., 2022). These functions are all related to membrane transport during virus replication in the silkworm, suggesting that in addition to proteins, ncRNAs may play an important role in the silkworm immune system following the inhibition of HSP90 (Zhao Z.-M. et al., 2022).

### 3.4. Circular RNA

CircRNA was first identified by using electron microscopy in 1976 but did not attract much attention then. Only in recent years, with the development of the high-throughput sequencing technique and the discovery of a series of circRNAs, researchers have gradually realized the specificity of circRNA and its functional importance *in vivo* (Sanger et al., 1976; Jeck et al., 2013; Memczak et al., 2013; Abbas et al., 2023). With the development of technology, the functions of circRNA have been found not only to act as a miRNA sponge that regulates the transcriptional level but also to encode proteins and regulate protein binding at different levels (Qu et al., 2015; Kristensen et al., 2019).

The circRNAs first found in silkworms were in 2017. Researchers discovered specifically expressed circRNAs in the silk glands, and a circRNA-miRNA network was constructed (Gan et al., 2017). Thirteen miRNAs interacted with 193 circRNAs were then identified in BmCPV-infected silkworms by Hu et al. In contrast, in 2018, Hu et al. also identified 353 DEcircRNAs in BmNPV-infected silkworms. GO and KEGG enrichment analysis for the target sites of miRNAs regulated by circRNAs showed ubiquitination, apoptosis, and endocytosis enriched in the silkworms (Gan et al., 2017; Zhang et al., 2022). The signaling pathways of the BmNPV-infected silkworms were determined, and these pathways could regulate the *Bombyx mori* immune pathways (Gan et al., 2017; Zhang et al., 2022). It was the same as the lncRNA, circRNA-miRNA-mRNA network also found in the silkworm fat body by Yin et al. (2021) and after performing GO and KEGG analysis, it was shown that the fat body is significantly involved in the immune system. These results demonstrated the important role of circRNA in viral infection for the first time.

**Figure 3:**
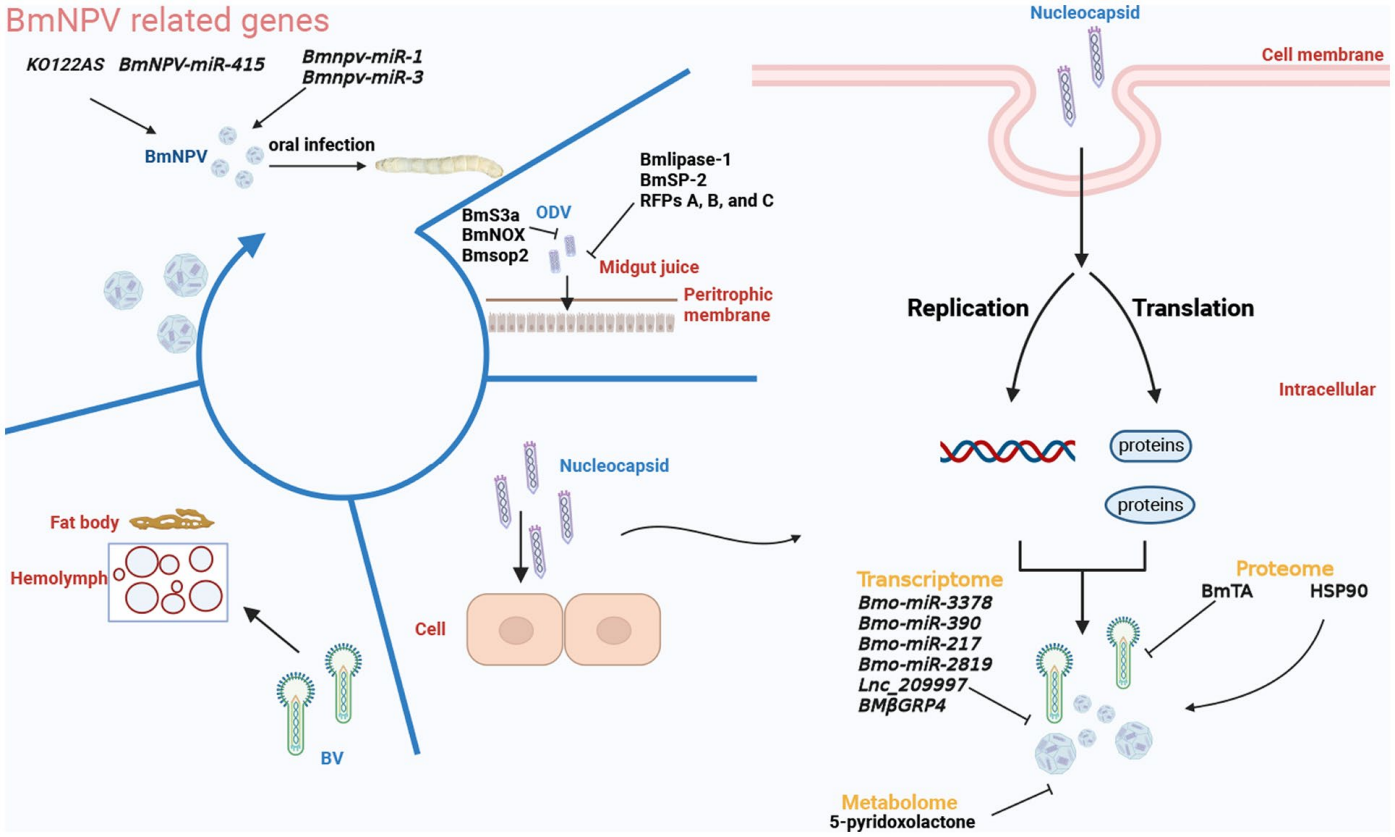
The replication of BmNPV is divided into several parts that are discontinuous in time. According to the existing omics research, the strong inhibitory genes, proteins, and metabolites are distinguished and shown in the figure. The figure was created with Biorender.com.

Although current circRNA research focuses mainly on circRNA as a miRNA sponge to regulate mRNA expression, some studies also demonstrated that circRNA regulated histone modification. Wang et al. found that the cyclized *BmEgg* gene in *B.mori* (gene cyclized by the eggless *B. mori* histone-lysine N-methyltransferase (*BmEgg*) gene), circEgg positively regulates histone deacetylase (HDAC) expression in *B. mori via* the sponge with *bmo-miR-3391* to inhibit the methylation of lysine 9 of histone H3 and promote acetylation of lysine 9 of histone H3 (Wang et al., 2020). Some sites were essential for the replication of the virus, like the lysine acetylation (Kac) sites, which were found in the late expression factor −3/−4/−6/−11 (*Lef-3/−4/−6/−11*). In previous studies, 4 Kac sites were required for late viral replication, suggesting the protein acetylation might have unsure functions in the viral replication (Rapp et al., 1998; Mikhailov et al., 2006; Yu and Carstens, 2012; Hu D. et al., 2018). Then in one Kac site, *Lef-3*, two acetylation modification sites, including K18 and K27, were located in the conserved *Lef-3* region. And the K18 acetylation site would cause the binding of *Lef-3* and *P143* (a DNA helicase protein) by the experiments, making it difficult to replicate the virus in BmN cells (Ito et al., 2004; Gao et al., 2021). Indirectly controlling the protein acetylation modification in infected silkworms through the regulation of miRNA-protein modifying enzymes caused the complex replication of BmNPV in non-resistant silkworms (Wang et al., 2020). These findings suggested that silkworms could be regulated by overexpressing or repressing circRNA to realize the resistance. We believe that by modifying the protein, noncoding RNAs may have a lower impact on the silkworm phenotype than other methods, such as directly knocking out some key genes, because their low expression, non-coding, and target genes are predictable, giving them the unique advantage of having little effect on other none-resistant genes or proteins. All related genes or substances are classified and shown in Figure 3.

## 4. Conclusion

Multi-omics studies have provided many research ideas for disease resistance in the domestic silkworm. However, the key resistant genes and their mechanisms are still unclear. It is still unknown why and how these differences arise and whether these DEGs are due to their sequences, such as exon loss or various modifications. And whether the DEPs themselves may be distinctly different due to SNPs or protein modifications remain unknown. However, with the discovery of multiple ncRNAs, DEGs, DEPs, and DSMs regulated by ncRNAs, circRNA (lncRNA)-miRNA-mRNA regulatory networks were discovered individually. These differences are not distinct from the previous results of studies, which included metabolism, membrane fusion, modification, immunity, and viral regulation. These intersections not only demonstrated an unidentified set of regulatory mechanisms of ncRNAs in the resistant silkworm but also explained that these DEGs, DEPs, and DSMs may be due to exogenous or endogenous ncRNAs. Non-coding RNAs researches are certainly a faster and more effective approach because a direct screen of a large number of possible disease-resistant genes based on the regulatory network (Wang J. et al., 2019; Wu et al., 2020; Ning et al., 2021; Sweeney et al., 2021), as they are widely present in animals and plants.

Recent studies on non-coding RNAs have shown that with the problem of silkworm BmNPV resistance, non-coding RNAs are likely to be regulated at the transcriptional level and ultimately affect protein expression. However, BmNPV resistance is a complex issue, and current studies can only identify the involvement of ncRNAs. Still, there is yet to be definitive literature proving that they play an irreplaceable role. At the same time, ncRNAs can explain why these DEGs, DEPs, and DSMs at the expression level, either by direct regulation of viral ncRNAs or by modification and regulation of host proteins with ncRNAs, which all resulted in these differences at the expression level and finally affected viral replication. However, ncRNAs have only recently been studied; the relationship between ncRNAs and BmNPV has only been investigated recently. No studies have shown the exact reasons why and what caused these ncRNAs to differ, whether they differ because there are unknown genes upstream to regulate them or whether their sequences undergo changes after viral infection. At the same time, existing studies have shown that it is not a kind of ncRNA in playing an important regulatory role, such as miRNA, piRNA, and siRNA. These three non-coding RNAs are very similar in length and function; from the perspective of bioinformatic analysis, more accurate algorithms need to be developed to achieve precise identification, and distinguish its source, is the foreign virus or the host itself in order to facilitate the development of subsequent experiments; on the other hand, viral ncRNA regulation of host ncRNA and genes has been demonstrated. And the BmNPV genome is much smaller than the size of the silkworm genome, which may be better for researchers to screen for resistance genes from the viral genome, but such studies are still rare. More experimental results are needed to verify this to help us understand precisely what happens after BmNPV infection and how these changes occur.

Right now, some research has been achieved for BmNPV resistance in silkworms, and various omics have also made the screening of resistance genes less problematic. But the final determination of which is the resistant gene is still a long-term research topic, and its specific mechanism cannot be analyzed by a single omics study alone. The screening of resistant genes in silkworms should be based on the ncRNAs regulatory network of multi-organismal and multi-omics in silkworms to finally obtain the key reliable resistant genes.

## Author contributions

Y-XF and LC: conceptualization. Y-XF, VA, and LC: methodology. Y-XF: resources, writing—original draft preparation, and visualization. LC and VA: writing—review and editing. LC: supervision, project administration, and funding acquisition. All authors have read and agreed to the published version of the manuscript.

## Funding

This work was funded by the National Natural Science Foundation of China, grant number 31802140.

## Conflict of interest

The authors declare that the research was conducted in the absence of any commercial or financial relationships that could be construed as a potential conflict of interest.

## Publisher’s note

All claims expressed in this article are solely those of the authors and do not necessarily represent those of their affiliated organizations, or those of the publisher, the editors and the reviewers. Any product that may be evaluated in this article, or claim that may be made by its manufacturer, is not guaranteed or endorsed by the publisher.
